# Epigenome-wide association study of objectively measured physical activity in peripheral blood leukocytes

**DOI:** 10.1186/s12864-025-11262-0

**Published:** 2025-01-22

**Authors:** Nicolas Fragoso-Bargas, Nancy S. Mcbride, Sindre Lee-Ødegård, Deborah A. Lawlor, Paul D. Yousefi, Gunn-Helen Moen, Julia O. Opsahl, Anne Karen Jenum, Paul W. Franks, Rashmi B. Prasad, Elisabeth Qvigstad, Kåre I. Birkeland, Kåre R. Richardsen, Christine Sommer

**Affiliations:** 1 Mohn Center for Diabetes Precision Medicine, Department of Clinical Science, University of Bergen, Bergen, Norway; 2 Department of Endocrinology, Morbid Obesity and Preventive Medicine, Oslo University Hospital, Oslo, Norway; 3 Institute of Clinical Medicine, Faculty of Medicine, University of Oslo, Oslo, Norway; 4 MRC Integrative Epidemiology Unit at the University of Bristol, Bristol, United Kingdom; 5 Population Health Science, Bristol Medical School, University of Bristol, Bristol, UK; 6 NIHR Bristol Biomedical Research Centre, University Hospitals Bristol and Weston NHS Foundation Trust and University of Bristol, Bristol, UK; 7 Institute of Molecular Bioscience, The University of Queensland, Brisbane, Australia; 8 Department of Public Health and Nursing, K.G. Jebsen Center for Genetic Epidemiology, NTNU, Norwegian University of Science and Technology, Trondheim, Norway; 9 The Frazer Institute, The University of Queensland, Woolloongabba, Australia; 10 General Practice Research Unit (AFE), Department of General Practice, Institute of Health and Society, Faculty of Medicine, University of Oslo, Oslo, Norway; 11 Lund University Diabetes Centre, Department of Clinical Sciences, Lund University, Malmö, Sweden; 12 Institute for Molecular Medicine Finland FIMM, Helsinki University, Helsinki, Finland; 13 Faculty of Health Sciences, Department of Rehabilitation Science and Health Technology, Oslo Metropolitan University, Oslo, Norway

**Keywords:** DNA methylation, Epigenetic epidemiology, EWAS, Physical activity, Moderate activity, Sedentary behavior, EPIPREG, ALSPAC, Pregnancy

## Abstract

**Background:**

Few studies have explored the association between DNA methylation and physical activity. The aim of this study was to evaluate the association of objectively measured hours of sedentary behavior (SB) and moderate physical activity (MPA) with DNA methylation. We further aimed to explore the association between SB or MPA related CpG sites and cardiometabolic traits, gene expression, and genetic variation.

**Results:**

For discovery, we performed cross sectional analyses in pregnant women from the Epigenetics in pregnancy (EPIPREG) sample with both DNA methylation (Illumina MethylationEPIC BeadChip) and objectively measured physical activity data (SenseWear™ Pro 3 armband) (European = 244, South Asian = 109). For EWAS of SB and MPA, two main models were designed: model (1) a linear mixed model adjusted for age, smoking, blood cell composition, including ancestry as random intercept, and model (2) which was additionally adjusted for the total number of steps per day. In model 1, we did not identify any CpG sites associated with neither SB nor MPA. In model 2, SB was positively associated (false discovery rate, FDR < 0.05) with two CpG sites within the *VSX1* gene. Both CpG sites were positively associated with BMI and were associated with several genetic variants in cis. MPA was associated with 122 significant CpG sites at FDR < 0.05 (model 2). We further analyzed the ten most statistically significant MPA related CpG sites and found that they presented opposite associations with sedentary behavior and BMI. We were not able to replicate the SB and MPA-related CpG sites in the Avon Longitudinal Study of Parents and Children (ALSPAC). ALSPAC had available objectively measured physical activity data from Actigraph (without steps/day available) and leucocyte DNA methylation data collected during adolescence (*n* = 408, European).

**Conclusion:**

This study suggests associations of objectively measured SB and MPA with maternal DNA methylation in peripheral blood leukocytes, that needs to be confirmed in larger samples of similar study design.

**Supplementary Information:**

The online version contains supplementary material available at 10.1186/s12864-025-11262-0.

## Introduction

The World Health Organization (WHO) states that physical inactivity is the fourth leading risk factor for global mortality [1]. Physical activity is associated with weight control [2], improved blood lipid profile [3] and insulin sensitivity [4], and decreased risk of cardiovascular disease and type 2 diabetes [5]. Substantial health benefits are seen after performing the recommended 150 min/week of moderate to vigorous physical activity [1, 6, 7]. However, the protective mechanisms of physical activity are not fully elucidated.

Physical activity has several impacts in the immune system. It reduces the production of pro-inflammatory cytokines and increases anti-inflammatory cytokines [8]. Furthermore, it increases the mobilization of leukocytes [9], and improves the immune system response [10]. Hence, physical activity may exert DNA methylation changes in blood leukocytes that could mediate some of its positive health effects [11]. Two cross-sectional epigenome-wide association studies have identified associations of questionnaire-reported physical activity from adult men and women with specific CpG sites [11, 12] in peripheral blood leukocytes. In the first of these studies (*N* = 619), it was reported that one CpG site was associated with total physical activity (*p* = 6 × 10^− 09^). In the second study (*n* = 1745) two CpG sites were related to moderate to vigorous physical activity (p = < 1.18 × 10^− 7^). Notably, neither of the mentioned studies attempted to replicate their findings in independent cohorts, and there was no overlap in the identified associations across the two studies. Self-reported PA may be misclassified and, because of that, result in biased associations [13]. A recent EWAS (*n* = 3567) reported the association between methylation and physical activity objectively measured with an activity monitor [14]. No CpG remained after correcting for multi-testing, but the authors reported seven CpG sites nominally associated (p = < 1 × 10^− 5^) with moderate-to‐vigorous activity and 12 related to total MET-hours.

Thus, the current literature has not identified robust CpG sites associated with physical activity. Furthermore, no study has used a cross-ancestry design, or explored associations between the identified sites and cardiometabolic phenotypes related to physical activity. Hence, the aims of this study were: (1) to perform EWAS of objectively measured moderate physical activity (MPA) and sedentary behavior (SBP) in peripheral blood leukocytes, (2) attempt replication of the identified CpG sites in an independent cohort, (3) attempt replication of previously published CpG sites in our cohort, (4) explore the association of PA related CpG sites with other cardiometabolic phenotypes, (5) determine if the methylation levels of selected CpG sites are associated with genetic variants and gene expression.

## Methods

### Study population

The Epigenetics in Pregnancy (EPIPREG) cohort [15] consists of all women with European (*n* = 312) or South Asian (*n* = 168) ancestry with available DNA samples that participated in the STORK Groruddalen (STORK G) study. STORK G is a population-based cohort of 823 pregnant women from the district of Groruddalen, Oslo, Norway (2008–2010), and has been described in detail previously [16]. In short, women were enrolled between 8 and 20 weeks of gestation and could communicate in Norwegian or any of eight translated languages. Women who had pre-existing diabetes or who required specialist care during their pregnancy were excluded. Ethnic origin was defined by the participant’s country of birth, or by her mother´s country of birth if the latter was born outside Europe. The study gathered data at inclusion (8–20 weeks), at gestational week 28, and 12-weeks post-partum.

### Measurements of physical activity

The SenseWear™ Pro3 armband (BodyMedia Inc, Pittsburgh, PA, USA) was used to measure physical activity [17] at approximately gestational week 28. Participants were asked during the study visit to wear it continuously for the next 4 to 7 days, also during sleep, except during shower/water activities. The armband data were downloaded and analyzed with software developed by the manufacturer (SenseWear Professional Research Software Version 6.1, BodyMedia Inc., Pittsburgh, Pennsylvania, USA). One valid day of physical activity was defined as ≥ 19.2 h of wear time [18]. The SenseWear™ Pro3 automatically detects non-wear time, increasing the accuracy of the measurements [19]. Women with at least two valid days were included in the analyses. From the armband data, we extracted number of steps, mean hours/day of moderate-intensity physical activity (MPA) (defined as ≥ 3.0–6.0 metabolic equivalents of task), and sedentary behavior (SB) (defined as < 1.5 metabolic equivalents of task) [17, 20]. We limited our analysis to moderate intensity activity as only three women reached vigorous activity for an extended period (> 20 min) and the majority lacked data (61%) (details in Supplementary Table 1). Furthermore, when attempting to combine moderate and vigorous activity, outliers difficult to control for EWAS were introduced.

### DNA methylation measurement and genotyping

Blood samples were collected in gestational week 28 and DNA extraction was performed using a salting out protocol [21] at the Hormone Laboratory, Oslo University Hospital. Both DNA methylation and genotyping were performed at the Department of Clinical Sciences, Clinical Research Centre, Lund University, Malmö, Sweden. Details about the methods have been described thoroughly in EPIPREG’s cohort profile [15].

DNA methylation was quantified in peripheral blood leukocytes with the Infinium Methylation EPIC BeadChip (Illumina, San Diego, California, USA). The Epic BeadChip measures the proportion of methylation at ~ 850k CpG sites, giving values from 0 to 1, with this measure conventionally referred as ‘beta-values’. Meffil R package [22] was used for quality control (QC). We removed 6 sample outliers from the methylated/unmethylated ratio (> 3SD), 1 outlier in bisulfite 1 and bisulfite 2 control probes (> 5 SD), and 1 sample with sex mismatch (predicted sex outlier > 5 SD). Since the cohort included only pregnant women, this is likely a technical error. We filtered probes with detection *p*-value < 0.01 and bead count < 3. Samples from 472 of the 480 subjects, and 864,560 probes passed the QC. To minimize potential technical variation, all samples were randomly distributed across the beadchips, Functional normalization as implemented in Meffil, was used to obtain normalized beta-values standardized for 10 principal components from the QC, and potential batch effects such as slide, row, and columns. We omitted probes harboring X and Y chromosomes, cross-reactive probes, and probes harboring single nucleotide polymorphisms (SNPs). A total of 792,530 probes were analyzed. The data has been technically validated previously, and both the EPIC bead chip and pyrosequencing showed good agreement [15]. Beta values that were three times the interquartile range below or above the 25^TH^ and 75^TH^ percentiles respectively were removed.

Genotyping was performed using the CoreExome chip (Illumina, San Diego, California, USA) This chip interrogates ~ 250k single nucleotides across the genome. PLINK 1.9 [23] was used for QC and variant filtering. Genetic variants that deviate from Hardy Weinberg equilibrium (*p* = 1.0 × 10^− 4^), with low call rate (< 95%), and with a minor allele frequency (MAF) < 1% were filtered. Genetic data from 300 Europeans and 138 South Asians passed the QC, and approximately 300,000 variants were used for imputation. The GWAS scaffold was mapped to the NCBI build 37 of the human genome. For each ancestry we used the correspondent 1000 genomes project reference panel (Phase 3, - http://www.well.ox.ac.uk/~wrayner/tools/) [24], using IMPUTE2 version 2.3.2 [25]. PLINK 1.9 was also used for post-imputation QC. We removed non-SNP variants and low-quality post-imputation SNPs (info < 0.9) and SNPs with MAF < 5%. Genetic ancestry from principal component analysis corresponded completely with self-reported ethnicity [15].

### Covariates

Age, smoking status and blood cell composition were considered as the main covariates for this study. Maternal age at enrollment was calculated from birth date. Smoking status was assessed with an interviewer-administered questionnaire and dichotomized into smokers (current and during the last three months before pregnancy) and nonsmokers (former and never smokers). Blood cell composition (CD8T, CD4T, NK, β-cells, monocytes, and neutrophils) was calculated with the Houseman´s method [26] with the Meffil R package [22].

### Assessment of phenotypes used for follow-up analyses

We had available data on education level (< or ≥ 10 years of education), BMI, glucose measurements (fasting and 2-hour glucose), GDM diagnosis (WHO 99 criteria), insulin measurements (insulin, C-peptide and HOMA-IR), total cholesterol, LDL-cholesterol, HDL-cholesterol, triglycerides, and both systolic and diastolic blood pressure. Details on the laboratory measurements are available in the Supplementary material, and can be consulted as well in EPIPREG’s cohort profile [15].

### Study flow

**Fig. 1:**
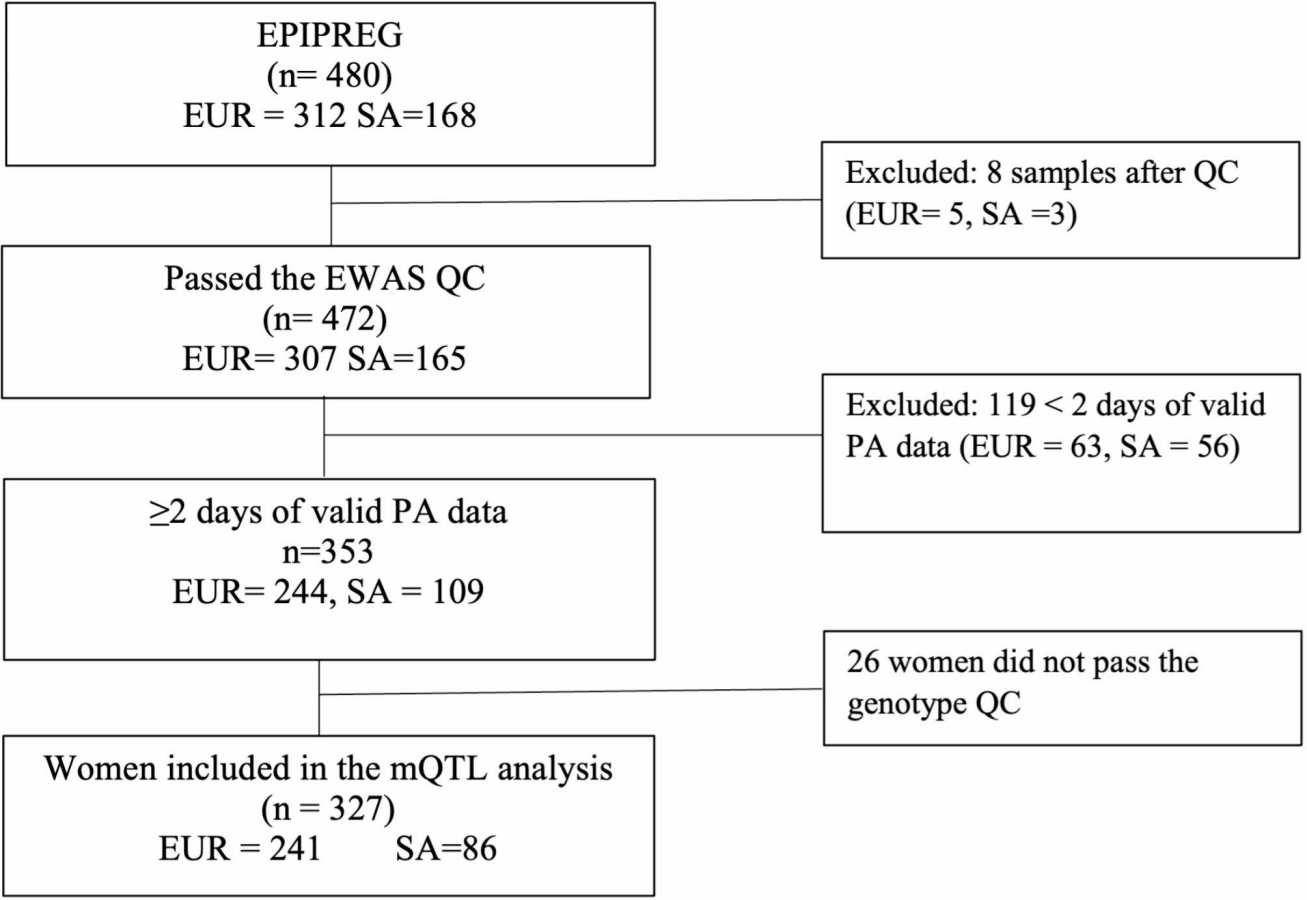
Overall study flow. EUR = European, SA = South Asian

Of the 480 women in EPIPREG, 472 passed the EWAS QC. From these, 353 samples (European = 244, South Asians = 109) had valid PA data (Fig. 1).

### Statistical analysis

#### Discovery analysis

Beta values were logit-transformed into M-values. For the EWAS analyses, we used a cross-ancestry approach to find common biologically relevant DNA methylation marks and to increase generalizability, as we have done successfully previously [27].

We designed two models for discovery analyses: (1) a linear mixed model testing the association between the two exposure variables (MPA and SB) and M-values (outcome) using the R packages lme4 [28] and lmerTest [29]. European or South Asian ancestry was included in the model as random intercepts, while age, smoking status, number of days using the armband, and estimated blood cell composition were included as fixed effects, (2) similar to model 1, but additionally controlling for the number of steps per day. We considered the total number of steps per day a proxy of general activity level [30], thus by controlling for it we isolate the effect of SB and MPA on DNA methylation.

*P*-values of the linear mixed model main effects were calculated with Satterthwaite’s method [29]. We used a false discovery rate (FDR) of 5% to account for multiple testing [31]. Findings with *p* < 1.0 × 10^− 4^, the threshold used in the EWAS catalog [32], are also reported. We used the QCEWAS R package [33] to calculate the inflation for each EWAS performed.

#### Variance inflation factor

For selected CpG sites, we evaluated whether the inclusion of the daily total number of steps as a covariate (model 2) introduced multi-collinearity. If the variance inflation factor (VIF) for each covariate was >5.0, we removed highly correlated variables (*r* > 0.7) from the model and recalculated VIF for each covariate if necessary. Lastly, we compared the coefficients and *p*-values towards the discovery results.

#### Associations with cardiometabolic traits and sensitivity analyses

To evaluate the associations between cardiometabolic traits and methylation of CpG sites selected for follow-up analyses we used linear mixed models, adjusting for blood cell composition, age, smoking, and ancestry as random intercepts. For these associations, we used an uncorrected two-sided *p* < 0.05 was accepted.

We also performed sensitivity analysis by adjusting for BMI, GDM, and education to evaluate its potential influence on the associations followed further.

#### Methylation Quantitative Trait Loci (mQTL) analysis

Cross-ancestry mQTL analyses were performed owing to the small sample size and under the assumption that genetic variants have similar effects across ancestries [27]. To minimize the computation burden, we used the GEM R package [34] separately in Europeans and South Asians, adjusting for age, smoking, and blood cell composition. The results were combined by using a custom fixed effect meta-analysis R script identical to the inverse variance-weighted average method implemented in METAL [35]. We used a standard GWAS *p*-value threshold of 5 × 10^− 8^ for mQTLs. cis-mQTLs were defined as located +-<1 Mb from the CpG, otherwise, they were classified as trans-mQTLs. We tested the most significant mQTL of each CpG site for association with several cardiometabolic phenotypes using linear mixed models with ancestry as random intercepts.

#### Gene expression analysis in MESA

The publicly available dataset from the Multi-Ethnic Study of Atherosclerosis (MESA) (*n* = 1202) (Gene Expression Omnibus, GSE56580) was used to test Spearman’s correlations between DNA methylation and gene expression in CD4 + cells [36]. The DNA methylation data was performed on the 450k platform.

#### Comparison with previously reported associations

We verified whether the significant CpG sites previously reported from questionnaire based data [11, 12], along with the nominal CpG sites reported for objectively measured physical activity [14] could be identified in our sample as well. As the methodology to define the measurements for physical activity differ within these studies and our own, we mainly looked up if a CpG site reached at least *p* < 0.05.

### Replication in an independent cohort

We used the Avon Longitudinal Study of Parents and Children (ALSPAC) cohort for replication (Details in Supplementary material). Briefly, pregnant women resident in the former county of Avon, in the South West of the UK with expected dates of delivery from 1st April 1991 to 31st December 1992, were recruited. The cohort includes 14,541 pregnancies, resulting in 14,676 fetuses, 14,062 live births, and 13,988 children who were alive at 1 year. When the children were 7 years old, efforts were made to recruit children in the area who had been born between the expected dates of the original recruitment period. This resulted in a total of 15,454 pregnancies, 15,589 fetuses, and 14,901 children who were still alive at 1 year, who have been followed into adulthood [37, 38]. Of note, the study website contains details of all the data that is available through a fully searchable data dictionary and variable search tool (http://www.bristol.ac.uk/alspac/researchers/our-data/).

For replication, we used the follow-up data of the offspring at ~ 15 years, of which only a subset had available DNA methylation data (*n* = 1001). The ~ 15 years old subset was chosen because it was the nearest age to adulthood in ALSPAC who had both PA and methylation data. We performed cross-sectional analyses of associations between PA related CpG sites identified in EPIPREG and objectively measured PA, without adjustment for steps/day. Objectively measured physical activity was measured with either the MTI Actigraph 7164 or 71,256 accelerometers (Actigraph LLC, Fort Walton Beach, FL, USA). Like the discovery analysis, only participants who had at least two valid days of physical activity were included in the replication analysis (*n* = 408). Details of the sample selection and population characteristics can be consulted in Supplementary Fig. 1 and Supplementary Table 2 respectively. Physical activity was classified as SB (0-100 counts per minute (cpm)), Moderate to Vigorous (MVPA) (> 2296 cpm), and Vigorous (> 4011 cpm). DNA methylation in peripheral blood leukocytes was quantified with the Illumina array (Infinium HumanMethylation450 BeadChip). For MPA related CpG sites, 76/121 CpG sites were available in the 450k beadchip and 1/2 SB related CpG sites.

Only model 1, without steps/day, was possible to perform in the replication sample, as steps/day was not collected in ALSPAC. We considered CpG sites as replicated if they had a consistent direction of the effect towards the discovery analysis and passed an FDR threshold of 5%. However, we also report CpG sites that were directionally consistent and had a *p*-value < 0.05.

## Results

### Population characteristics

The characteristics of the women included in this study are presented in Table 1. The mean age was 30.0 years (SD = 4.6). On average, participants engaged in 17.9 h per day (SD = 1.6) of sedentary behavior and 1.1 h per day (SD = 0.8) of moderate physical activity. They averaged 8056.7 steps per day (SD = 3068.5).

**Table 1:** Characteristics of the sample. Numbers are mean (SD) or n (%)

Variable	*N*	All (*n* = 353)	*N*	European (*n* = 244)	*N*	South Asian (*n* = 109)
Age (years)	353	30.0 (4.6)	244	30.7 (4.5)	109	28.5 (4.5)
Gestational week	353	28.1 (1.2)	244	28.1 (1.1)	109	28.1 (1.2)
Nonsmokers	353	274 (78.1)	244	167 (69.0)	109	107 (98.2)
Smokers at week 28	17	17 (4.8)	17	17 (7.0)	0	0 (0.0)
Smokers 3 months pre-pregnancy	61	61 (17.3)	59	59 (24.2)	< 5	< 5 (< 5)
Former smokers	77	77 (21.8)	71	71 (29.1)	6	6 (5.5)
Never smokers	198	198 (56.1)	97	97 (39.7)	101	101 (92.7)
Less than 10 years of education	353	32 (9.1)	244	11 (4.5)	109	21 (19.4)
SB (hours/day)	353	17.9 (1.6)	244	18.0 (1.6)	109	17.7 (1.7)
MPA (hours/day)	353	1.1 (0.8)	244	1.2 (0.7)	109	1.0 (0.9)
BMI (kg/m^2^)	352	27.2 (4.6)		27.5 (4.8)		26.6 (4.3)
Number of steps/day	353	8056.7 (3068.5)	244	8477.1 (3012.8)	109	7115.78 (2995.3)
Hours of sleep	353	7.1 (1.0)	244	7.1 (0.9)	109	7.0 (1.3)
Fasting plasma glucose (mmol/l)	353	4.8 (0.6)	244	4.7 (0.6)	109	5.0 (0.6)
2-hour plasma glucose (mmol/l)	353	6.1 (1.4)	244	6.0 (1.4)	109	6.4 (1.5)
GDM WHO 1999 (yes)	353	41 (11.7)	244	26 (10.7)	109	15 (13.8)
C-peptide (pmol/l)	349	807.4 (341.5)	241	777.5 (347.9)	108	873.6 (318.4)
Insulin (pmol/l)	349	63.1 (39.3)	241	56.1 (36.3)	108	78.6 (41.4)
HOMA-IR	349	1.7 (0.8)	241	1.6 (0.8)	108	1.9 (0.7)
Triglycerides (mmol/l)	353	2.0 (0.7)	244	1.9 (0.7)	109	2.0 (0.6)
Total cholesterol (mmol/l)	353	6.2 (1.1)	244	6.4 (1.1)	109	6.0 (1.0)
HDL-cholesterol(mmol/l)	353	1.9 (0.4)	244	1.9 (0.4)	109	1.8 (0.4)
LDL-cholesterol(mmol/l)	349	3.5 (1.0)	241	3.6 (1.0)	108	3.3 (0.8)
Systolic blood pressure (mmHg)	349	104.7 (9.7)	243	106.6 (9.5)	106	100.4 (8.6)
Diastolic blood pressure (mmHg)	349	66.8 (6.8)	243	67.5 (6.8)	106	65.0 (6.5)

### Associations between objectively measured physical activity and DNA methylation

**Fig. 2:**
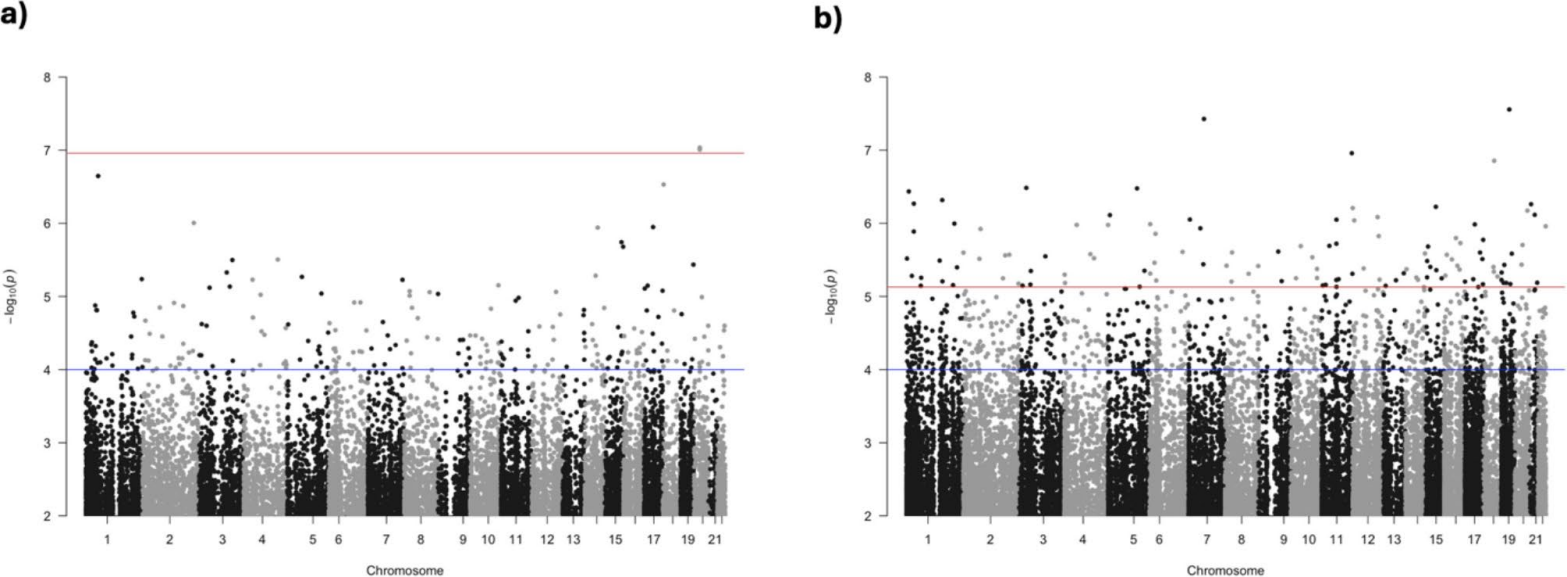
Manhattan plots for the EWAS of SB (**a**) and MPA (**b**) adjusted for steps/day. In the SB EWAS (**a**) we identified two CpG sites associated with hours of SB. For the MPA EWAS (**b**), we identified 122 CpG sites associated with hours of MPA. The blue lines denote *p* < 1 × 10^− 4^, and the red lines denote an FDR < 0.05

In model 1 we did not identify any CpG sites associated with SB (λ = 1.06) or MPA (λ = 1.15) with FDR < 0.05 (Supplementary Tables 3 and 4). In model 2, adjusted for number of steps/days, we identified two CpG sites associated with SB (λ = 1.25) and 122 with MPA (λ = 1.27) that passed the FDR threshold (Fig. 2; Table 2). The CpG sites with *p* < 1.x10^− 4^ from model 2 are available in Supplementary Tables 5 and 6 for the hours of SB and MPA EWAS, respectively. The *p*-value QQ-plots for each EWAS can be consulted in Supplementary Figs. 2 and 3.

### Replication analysis in an independent cohort

As we only obtained significant CpG sites in model 2 adjusted for steps, we attempted replication of SB and MPA related sites in ALSPAC, despite not having steps/day. We did not find any CpG site that passed an FDR < 0.05 in any of the replication analyses (Supplementary Tables 7–9). In the MVPA analysis (Supplementary Table 8), only cg12424475 (in *ANKRD35*) had *p* < 0.05 (Effect = -0.072, SE = 0.025), and a consistent direction of the effect with the MPA analysis in EPIPREG (Effect = -0.126, SE = 0.027).

### Comparison with previously reported associations

cg10266336, associated with total physical activity assessed with questionnaires [11], was not available in our data. The two CpG sites associated with MVPA assessed with questionnaire [12], cg24155427 and cg09565397, did not reach *p* < 0.05 in our sample and the authors did not report the coefficients (Supplementary Table 10). Of the seven nominally associated CpG sites associated with objectively measured MVPA (Supplementary Table 10), none reached *p* < 0.05. Of the 12 CpG sites nominally associated with total MET-hours (Supplementary Table 10), only cg17385847 (in the *NPM1* gene) was associated with MPA in EPIPREG in model 2 (Effect: 0.105, SE: 0.050).

### CpG sties selected for further analyses and associations across physical activity variables

For follow up analysis we focused on the two SB associated CpG sites, and the top ten most significant MPA related CpG sites, due to possible inflation and lack of replicated sites (Table 2). One of the SB related CpG sites (cg26698820) had an opposite direction of effect (*p* < 0.05) compared to the same site in relation to MPA (*p* < 0.05) (Table 2). Eight of the ten MPA-related CpG sites with the lowest *p*-value displayed opposite effect sizes (*p* < 0.05) when contrasted with SB (Table 2).

**Table 2:** Associations across physical activity variables and BMI of the ten most significant MPA related CpG and the two CpG sites associated with hours of sedentary behavior. All data are from model 2 (adjusted for steps)

**CpG sites significantly associated with sedentary behaviours**								
**CpG information**			**SB**		**MPA**		**BMI**	
**CpG (Gene)**	**Chr**	**Relation to transcription**	Effect(SE)*	***p*** **-value**	Effect(SE)*	***p*** **-value**	Effect(SE)*	***p*** **-value**
cg26698820 (*VSX1*)	20	Near promoter	-0.056 (0.010)	9.22 × 10^− 8^	0.078 (0.024)	0.001	-0.010 (0.003)	0.001
cg19592637 (*VSX1*)	20	Near promoter	-0.067 (0.013)	9.82 × 10^− 8^	0.039 (0.029)	0.184	-0.010 (0.004)	0.009
**Top ten CpG sites significantly associated with moderate physical activity**								
**CpG information**			**SB**		**MPA**		**BMI**	
**CpG (Gene)**	**Chr**	**Relation to transcription**	Effect* (SE)	***p*** **-value**	Effect* (SE)	***p*** **-value**	Effect* (SE)	***p*** **-value**
cg02014733 (*WTIP*)	19	Body	0.020 (0.009)	0.024	-0.107 (0.019)	2.78 × 10^− 8^	0.003 (0.003)	0.171
cg12482933 (N/A)	7		-0.012 (0.005)	0.015	0.060 (0.011)	3.74 × 10^− 8^	-0.004 (0.001)	0.005
cg08327999 (N/A)	11		-0.011 (0.01)	0.272	0.116 (0.021)	1.10 × 10^− 7^	-0.005 (0.003)	0.056
cg05094046 (*DYM*)	18	Near promoter	0.012 (0.008)	0.152	-0.095 (0.018)	1.40 × 10^− 7^	0.003 (0.002)	0.186
cg13264633 (N/A)	3		-0.022 (0.010)	0.031	0.119 (0.023)	3.28 × 10^− 7^	-0.007 (0.003)	0.027
cg17943107 (N/A)	5	Body	-0.023 (0.008)	0.004	0.089 (0.017)	3.33 × 10^− 7^	-0.006 (0.002)	0.004
cg11949866 (*CASZ1*)	1	5’ UTR	0.031 (0.015)	0.046	-0.176 (0.034)	3.67 × 10^− 7^	0.011 (0.004)	0.016
cg15934095 (N/A)	1		-0.041 (0.011)	3.2 × 10^− 4^	0.129 (0.025)	4.81 × 10^− 7^	-0.009 (0.003)	0.006
cg07919197 (*TXLNA*)	1	Body	0.037 (0.013)	0.003	-0.141 (0.027)	5.39 × 10^− 7^	0.003 (0.004)	0.373
cg26085473 (*BTG3*)	21	Near promoter	0.039 (0.014)	0.004	-0.153 (0.030)	5.48 × 10^− 7^	0.010 (0.004)	0.014

*The effect size units are M-values

### Addressing potential multi-collinearity

For the CpG sites selected for further analysis, we evaluated the correlation within all the covariates included in model 2. Both CD4T-cells and CD8T-cells were highly correlated (*r* > 0.7) with Neutrophils, and the VIF was > 5 on all the white blood cell types included in the model. We further removed both CD4T and CD8T to evaluate the robustness of the associations. In this model, the VIF factor was < 5 for all the covariates included. The effect sizes and *p*-values changed marginally in comparison to the discovery analysis (Supplementary Table 11).

### Associations with cardiometabolic related traits

Among the ten CpG sites linked to MPA that we chose to investigate further, six showed significant associations with BMI (*p* < 0.05), as did both CpG sites linked to SB (Table 2). The SB-related CpG sites, which were positively associated with SB hours, were also positively associated with BMI. In contrast, six of the ten MPA-related CpG sites exhibited opposite effect sizes when associated with BMI (Table 2). No CpG site was associated with any other cardiometabolic trait examined (Supplementary Table 12).

### Sensitivity analyses

We further adjusted for BMI, GDM, and education to evaluate their influence on the selected CpG sites associated with MPA and SB. By adjusting for BMI, the direction of the effect sizes persisted, and the *p*-values were slightly attenuated but remained < 0.05 (Supplementary Table 13). The effect sizes and *p*-values changed marginally when adjusting for GDM (Supplementary Table 13). Lastly, in the analysis adjusted for education, half of the CpG sites had marginal changes while in the other half the direction of the effect was maintained but with slightly attenuated *p*-values (< 0.05) (Supplementary Table 13).

### Identification of mQTLs

Methylation at cg26698820 and cg19592637, which are associated with SB, was related to several SNPs (Table 3 and Supplementary Table 14). Their most significant SNPs, rs55651034 and rs6076315 respectively, showed an increased methylation effect at both CpG sites (Table 3). However, both SNPs were not associated with any of the tested cardiometabolic phenotypes (Supplementary Table 15). We did not identify any mQTL for the top 10 MPA-associated CpG sites.

**Table 3:** Most significant mQTLs associated with methylation at cg26698820 and cg19592637

CpG info			mQTL results		Most significant mQTL				
CpG	Chr	Gene	Number of cis-mQTL	Number of trans- mQTL	SNP (AL/MA)*	Chr	Gene	Effect (SE)	*p*-value
cg26698820	20	*VSX1*	99	0	rs55651034 (A > C)	20	*VSX1*	0.22 (0.02)	5.34 × 10^− 26^
cg19592637	20	*VSX1*	29	1	rs6076315 (G > A)	20	N/A	0.16 (0.03)	7.35 × 10^− 9^

*AL: Alternative allele, MA: Minor allele

### Associations with gene expression

Associations from the publicly available MESA data in CD4 + cells showed that methylation at cg05094046 is negatively correlated (rho = -0.11) with gene expression at *DYM* (Table 4). Methylation levels of cg11949866 and cg07919197 were positively correlated with gene expression at *CASZ1* (rho = 0.14) and *TXLNA* (rho = 0.10), respectively (Table 4).

**Table 4:** Associations between DNA methylation and gene expression. Both methylation and expression data are from CD4 + cells (MESA, *n* = 1202). Only CpG sites common in both 450k and EPIC platforms were analyzed

CpG	Gene	Relation to transcription	Rho	*p*-value
cg05094046	*DYM*	Near promoter	-0.11	1.02 × 10^− 4^
cg11949866	*CASZ1*	5’UTR	0.14	2.3 × 10^− 07^
cg07919197	*TXLNA*	Body	0.10	4.67 × 10^− 4^
cg02014733	*WTIP*	Body	-0.035	0.222
cg19592637	*VSX1*	Near promoter	-0.022	0.453

## Discussion

We performed EWAS of objectively measured physical activity using methylation data from peripheral blood leukocytes in a cross-ancestry sample of pregnant women. We identified two CpG sites associated with SB and 122 CpG sites associated with MPA after controlling for the number of steps/day in addition to observed confounders, suggesting that these CpG sites are related to SB and MPA, independent of general physical activity. The majority of these CpG sites examined for further analysis (*n* = 10 for MPA, and *n* = 2 for SB) were associated with BMI, with the same direction in relation to SB and in the opposite direction for MPA. Both SB related CpG sites were located in *VSX1* and were associated with genetic variants in cis. Three of the examined MPA related sites, cg05094046, cg11949866, and cg07919197, were associated with gene expression in CD4 + cells (MESA, *n* = 1202). Findings were not replicated in adolescents with Actigraph data in an independent cohort (ALSPAC).

Our model adjusting for steps per day was designed to isolate the specific effect of SB and MPA on DNA methylation independently of general physical activity level. The reasoning for doing this was based on previous studies that suggest that SB is associated with increased risk of mortality and morbidity when adjusting for moderate to vigorous physical activity [39, 40]. Given this background, we hypothesized that adjustment for number of steps [30], we could estimate the specific effect of SB and MPA on DNA methylation independent of general physical activity level. The emergence of the associations we observed could be interpreted as potential effects of SB and MPA independent of general activity. However, we acknowledge that these results could also be explained by bias due to inflation, as suggested by the elevated lambda. Furthermore, although the number of steps may not causally alter DNA methylation levels, collider bias cannot be ruled out completely and could potentially be a source of bias. This is because steps per day may be a potential mediator and not a confounder, meaning that there could be unknown confounders between steps per day and DNA methylation that could introduce bias [41].

In comparison to previous studies, we explored replication in an independent cohort (ALSPAC) ALSPAC. It used an earlier DNA methylation chip which covered fewer CpG sites in comparison to the iteration used in our discovery sample. Of the 123 discovered CpG sites, 76 could be explored for replication, and we did not find evidence of it. This might be because some of our discovery results are false positives, underlying differences between the two study populations (EPIPREG consisted of adult pregnant women while ALSPAC consisted of adolescents of both sexes), or because we could not adjust for steps per day in ALSPAC. In our attempt to verify previously identified PA related CpG sites from published studies, only cg17385847 (*NPM1)* from the EWAS of objectively measured PA [14] had *p* < 0.05 in EPIPREG. The scarcity of associations could be due to false positives or underlying methodological differences across studies, such as questionnaire assessed vs. objectively measured PA, different statistical models, or different categorization of PA. Furthermore, the objective measured physical activity findings of Fox and collaborators [14] did not reach genome-wide significance which increases the risk of false positives. Lastly, these studies included both men and women, while our population consisted of pregnant women.

The inclusion of the total daily number of steps as a covariate did not affect the VIF scores, but the calculated blood cell composition introduced multi-collinearity in the model. Although it is recommended to adjust for the major blood cell types in peripheral blood leukocytes, it is known that the Houseman method to calculate white blood cell composition indeed could introduce multi-collinearity [42]. However, by excluding CD4T and CD8T, which were highly correlated with Neutrophils in our data, the VIF scores improved, and the coefficients changed marginally. Hence, the inclusion of the six major cell types in the model is unlikely to affect the conclusions. Another important possible bias is that physical activity was recorded after blood sample collection and GDM diagnosis, which could have affected the women’s physical activity patterns, i.e. being more active after diagnosis. However, adjustment for GDM in sensitivity analyses suggested that GDM had little impact on the reported associations.

Among the CpG sites selected for further analyses, the effect sizes for six of the ten MPA related CpG sites were inversely related to SB and BMI. In contrast, the effect directions for the SB related CpG sites and BMI were consistent. These relationships follow a trend similar to that of other studies where MVPA is usually negatively associated with BMI [43–45], while SB is positively associated with BMI [44].

Some of the examined CpG sites were in genes previously related to cardiometabolic health. Both SB related CpG sites (cg26698820 and cg19592637) were located in *VSX1*, a gene previously identified in a HbA1c GWAS [46]. Among the MPA related CpG sites, cg05094046 (*DYM*) and cg11949866 (*CASZ1*) lay in genes important for the homeostasis of the cardiovascular system. *CASZ1* is an essential gene for cardiac development [47], and loss of function mutation in this gene is associated with hereditary dilated cardiomyopathy [47]. Moreover, genetic variants in *CASZ1* have been associated with LDL cholesterol [48], total cholesterol [48], ischemic stroke [49], and both systolic and diastolic blood pressure [48]. *DYM* has genetic variants previously associated with body fat percentage [50], and coronary artery disease [51]. Methylation of cg05094046 (*DYM*) and cg11949866 (*CASZ1*) were associated with gene expression in MESA, implying that the methylation changes induced by MPA have transcriptional implications. However future studies are needed to replicate these associations and to evaluate whether these potential changes in methylation are long-term.

Among the MPA related CpG sites, cg07919197 was located in a gene previously associated with exercise (*TXLNA*) [52]. *TXLNA* codes for the cytokine interleukin 14, which plays a role in B-cell proliferation and antibody formation [53]. Kisma and collaborators [52] observed that *TXLNA’s* expression varies during exercise, as it increased immediately post intense exercise, but decreased 15 min after recovery. Although the conclusions of this study should be taken with caution as the total sample size was very small (*n* = 3), methylation at cg07919197 was associated with gene expression in *TXLNA*. The last hints that physical activity modifies *TXLNA* expression through DNA methylation changes.

Lastly, we used blood to explore associations because it is considerable easier to obtain than other tissues. Ideally, key tissues like skeletal muscle and adipose tissue would provide a more comprehensive view of DNA methylation and physical activity. However, these tissues are harder to collect in medium to large epidemiological studies due to their invasive nature. Future research should assess if our findings apply to tissues beyond blood.

Major strengths of this study are the well-characterized, population-based cohort, objectively recorded physical activity, and inclusion of two ancestries. Another strength is the availability of genetic data in our sample, which allowed us to perform mQTL analysis. Important limitations include the limited sample size. The DNA methylation quantification in EPIPREG was done in peripheral blood leukocytes while the expression analysis in MESA was only done in isolated CD4+, hence there could be differences in gene expression across studies due to differences in white blood cell composition. The inflation presented in model 2 could be indicative of potential false positives. Lastly, we lack a replication cohort with similar cohort characteristics and data to verify our findings.

In conclusion, we identified two cross-ancestry CpG sites associated with SB and 122 for MPA. Our findings provide a valuable foundation for future research aimed at understanding the epigenetic mechanisms underlying physical activity and its potential impact on health.

## Electronic supplementary material

Below is the link to the electronic supplementary material.

Supplementary Material 1

Supplementary Material 2

## Data Availability

The complete summary statistics of each EWAS are available in Zenodo (10.5281/zenodo.14658142).Due to strict regulations for genetic data and privacy protection of patients in Norway, all requests for data access are processed by the STORK Groruddalen project’s steering committee. Researchers can request access to the data by contacting the PI of STORK Groruddalen (a.m.l.brand@medisin.uio.no) or the PI of EPIPREG (christine.sommer@medisin.uio.no).The ALSPAC study website (http://www.bristol.ac.uk/alspac/researchers/our-data/) contains details of all the data that are available through a fully searchable data dictionary and variable search tool. The data used for this study can be accessed upon request by contacting the ALSPAC executive committee at ALSPAC-exec@bristol.ac.uk.The expression microarray data used in this study that belong to the MESA cohort, have been previously made publicly available in the Gene Expression Omnibus (GEO), and are accessible through accession number GSE56580.
