# Correction: Epigenome-wide association study of objectively measured physical activity in peripheral blood leukocytes

**DOI:** 10.1186/s12864-025-11311-8

**Published:** 2025-02-13

**Authors:** Nicolas Fragoso-Bargas, Nancy S. Mcbride, Sindre Lee-Ødegård, Deborah A. Lawlor, Paul D. Yousefi, Gunn-Helen Moen, Julia O. Opsahl, Anne Karen Jenum, Paul W. Franks, Rashmi B. Prasad, Elisabeth Qvigstad, Kåre I. Birkeland, Kåre R. Richardsen, Christine Sommer

**Affiliations:** 1 Mohn Center for Diabetes Precision Medicine, Department of Clinical Science, University of Bergen, Bergen, 5023 Norway; 2 Department of Endocrinology, Morbid Obesity and Preventive Medicine, Oslo University Hospital, Oslo, Norway; 3 Institute of Clinical Medicine, Faculty of Medicine, University of Oslo, Oslo, Norway; 4 MRC Integrative Epidemiology Unit, University of Bristol, Bristol, UK; 5 Population Health Science, Bristol Medical School, University of Bristol, Bristol, UK; 6 NIHR Bristol Biomedical Research Centre, University of Bristol, Bristol, UK; 7 Institute of Molecular Bioscience, The University of Queensland, Brisbane, Australia; 8 Department of Public Health and Nursing, K.G. Jebsen Center for Genetic Epidemiology, NTNU, Norwegian University of Science and Technology, Trondheim, Norway; 9 The Frazer Institute, The University of Queensland, Woolloongabba, Australia; 10 Department of General Practice, Institute of Health and Society, Faculty of Medicine, General Practice Research Unit (AFE), University of Oslo, Oslo, Norway; 11 Department of Clinical Sciences, Lund University Diabetes Centre, Lund University, Malmö, Sweden; 12 Institute for Molecular Medicine Finland FIMM, Helsinki University, Helsinki, Finland; 13 Faculty of Health Sciences, Department of Rehabilitation Science and Health Technology, Oslo Metropolitan University, Oslo, Norway

**Correction: BMC Genomics 26, 62 (2025)**

10.1186/s12864-025-11262-0

Following publication of the original article it was reported that there was an error in the affiliations due to a missing affiliation. The missing affiliation was “Population Health Science, Bristol Medical School, University of Bristol, Bristol, UK”, which is currently affiliation 5.

This affiliation has been added and the numbering has been corrected in the affiliations shown in this Correction. The previous affiliation listing is given below, and the original article has been updated.

The wrongly given affiliations were:

Nicolas Fragoso-Bargas^1,2,3^*, Nancy S. Mcbride^4,5^, Sindre Lee-Ødegård^2,3^, Deborah (A) Lawlor^4,5^, Paul D. Yousefi^4,5,6^, Gunn-Helen Moen^3,7,8,9^, Julia O. Opsahl^3^, Anne Karen Jenum^10^, Paul W. Franks^11^, Rashmi (B) Prasad^11,12^, Elisabeth Qvigstad^2,3^, Kåre I. Birkeland^2,3^, Kåre R. Richardsen^12^ and Christine Sommer^2^.

1 Mohn Center for Diabetes Precision Medicine, Department of Clinical Science, University of Bergen, Bergen 5023, Norway.

2 Department of Endocrinology, Morbid Obesity and Preventive Medicine, Oslo University Hospital, Oslo, Norway.

3 Institute of Clinical Medicine, Faculty of Medicine, University of Oslo, Oslo, Norway.

4 MRC Integrative Epidemiology Unit, University of Bristol, Bristol, UK.

5 NIHR Bristol Biomedical Research Centre, University of Bristol, Bristol, UK.

6 Institute of Molecular Biosciences, The University of Queensland, Brisbane, Australia.

7 Department of Public Health and Nursing, K.G. Jebsen Center for Genetic Epidemiology, NTNU, Norwegian University of Science and Technology, Trondheim, Norway.

8 The Frazer Institute, The University of Queensland, Woolloongabba, Australia.

9 Department of General Practice, Institute of Health and Society, Faculty of Medicine, General Practice Research Unit (AFE), University of Oslo, Oslo, Norway.

10 Department of Clinical Sciences, Lund University Diabetes Centre, Lund University, Malmö, Sweden.

11 Institute for Molecular Medicine Finland FIMM, Helsinki University, Helsinki, Finland.

12 Faculty of Health Sciences, Department of Rehabilitation Science and Health Technology, Oslo Metropolitan University, Oslo, Norway.

