# Supplementary material for: Epigenome-wide association study of objectively measured physical activity in peripheral blood leukocytes
Source: BMC Genomics. 2025 Jan 22;26:62. doi: 10.1186/s12864-025-11262-0 (PMC11755845; doi:10.1186/s12864-025-11262-0)
Supplement: Supplementary file 2 — Supplementary Material 2 [file 12864_2025_11262_MOESM2_ESM.docx]

**Supplementary methods**

***Measurement of phenotypes used for follow-up analyses***

To evaluate the level of education, the women were asked to report their highest level of education in nine categories, which were dichotomized into < 10 years of education (from primary education to lower secondary education) and ≥10 years of education (high school or higher-level education). All data was gathered at gestational week 28. We calculated the body mass index (BMI) of the women by measuring weight (Tanita-BC 418 MA) and height with a fixed stadiometer (1). For GDM diagnosis, overnight fasting venous blood was drawn into ethylenediaminetetraacetic acid (EDTA) tubes at gestational week 28. All women performed a 75g oral glucose tolerance test. Fasting and 2-hour glucose were measured with a validated point-of-care device (HemoCue, Angelholm, Sweden) (2). GDM was classified during the study using the WHO 1999 criteria (fasting plasma glucose ≥ 7.0 mmol/L or 2-hour plasma glucose ≥ 7.8 mmol/L).

Overnight fasting insulin and C-peptide were measured with non-competing immunofluorometric assays (DELFIA, PerkinElmer Life Sciences, Wallac Oy, Turku, Finland) (3). Calculation of the Homeostasis model assessment of insulin resistance (HOMA-IR) (3) was estimated with fasting glucose and C-peptide, using the HOMA2 calculator version 2.2.2 (<https://www.dtu.ox.ac.uk/homacalculator>) (4). Fasting total cholesterol, high-density lipoprotein (HDL) cholesterol, and fasting triglycerides were measured with a colorimetric method (Vitros 5.1 fs, Ortho clinical diagnostics, Neckargemünd, Germany) (1). We used Friedewald´s formula to calculate low-density lipoprotein (LDL) cholesterol levels (5).

The anthropometric data was measured at gestational week 28. Systolic and diastolic blood pressure was measured, at 28 weeks gestation, with the blood pressure monitor M6 Comfort HEM-7000-E (Omron, Kyoto, Japan) (6).

***The Avon Longitudinal Study of Parents and Children cohort***

We used the Avon Longitudinal Study of Parents and Children (ALSPAC) cohort for replication. ALSPAC recruited Pregnant women resident in the former county of Avon, in the South West of the UK with expected dates of delivery from 1st April 1991 to 31st December 1992. The initial number of pregnancies enrolled was 14,541. Of these initial pregnancies, there was a total of 14,676 fetuses, resulting in 14,062 live births and 13,988 children who were alive at 1 year of age. When the children were aged ~7 attempts were made to increase the sample size by recruiting all children in the area who had been born between the expected dates of the original cohort, with research-level data collected on those new children and some of their mother’s pregnancy data by health record linkage and maternal questionnaires. This results in a total of 15,454 pregnancies, resulting in 15,589 fetuses and 14,901 children who were still alive at 1 year, who have been followed now into adulthood. Further details on the recruitment and assessments have been published (7, 8).

ALSPAC does not have information on objectively measured physical activity in pregnancy in the initial recruited women. For replication, we performed cross-sectional analyses of associations of accelerometer-measured physical activity with DNA methylation data in peripheral blood leukocytes from adolescents (~15 years). The ~15-year follow-up assessment was chosen because this was the oldest age (so most similar to discovery participants) at which both accelerometer and DNA methylation data were available. Physical activity data was measured either with MTI Actigraph 7164 or 71256 accelerometers (Actigraph LLC, Fort Walton Beach, FL, USA), with participants asked to wear these on the right hip for seven days. As for the discovery analysis, only participants who had at least two valid days of physical activity were included in the replication analysis (N=408) (supplementary figure 2).

**References**

1. Sommer C, Sletner L, Morkrid K, Jenum AK, Birkeland KI. Effects of early pregnancy BMI, mid-gestational weight gain, glucose and lipid levels in pregnancy on offspring's birth weight and subcutaneous fat: a population-based cohort study. BMC Pregnancy Childbirth. 2015;15:84.

2. Fragoso-Bargas N, Opsahl JO, Kiryushchenko N, Böttcher Y, Lee-Ødegård S, Qvigstad E, et al. Cohort profile: Epigenetics in Pregnancy (EPIPREG) – population-based sample of European and South Asian pregnant women with epigenome-wide DNA methylation (850k) in peripheral blood leukocytes. PLOS ONE. 2021;16(8):e0256158.

3. Morkrid K, Jenum AK, Sletner L, Vardal MH, Waage CW, Nakstad B, et al. Failure to increase insulin secretory capacity during pregnancy-induced insulin resistance is associated with ethnicity and gestational diabetes. Eur J Endocrinol. 2012;167(4):579-88.

4. Mørkrid K, Jenum AK, Sletner L, Vårdal MH, Waage CW, Nakstad B, et al. Failure to increase insulin secretory capacity during pregnancy-induced insulin resistance is associated with ethnicity and gestational diabetes. European Journal of Endocrinology. 2012;167(4):579-88.

5. Waage CW, Mdala I, Stigum H, Jenum AK, Birkeland KI, Shakeel N, et al. Lipid and lipoprotein concentrations during pregnancy and associations with ethnicity. BMC Pregnancy Childbirth. 2022;22(1):246.

6. Waage CW, Mdala I, Jenum AK, Michelsen TM, Birkeland KI, Sletner L. Ethnic differences in blood pressure from early pregnancy to postpartum: a Norwegian cohort study. J Hypertens. 2016;34(6):1151-9.

7. Boyd A, Golding J, Macleod J, Lawlor DA, Fraser A, Henderson J, et al. Cohort Profile: The ‘Children of the 90s’—the index offspring of the Avon Longitudinal Study of Parents and Children. International Journal of Epidemiology. 2012;42(1):111-27.

8. Fraser A, Macdonald-Wallis C, Tilling K, Boyd A, Golding J, Davey Smith G, et al. Cohort Profile: the Avon Longitudinal Study of Parents and Children: ALSPAC mothers cohort. Int J Epidemiol. 2013;42(1):97-110.

**Supplementary figures**

**
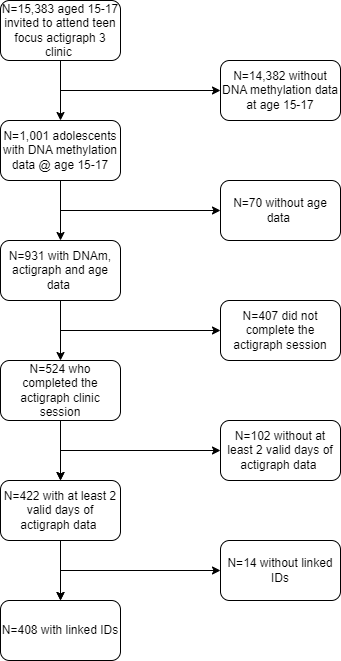
**

**Supplementary figure 1**: Flow diagram which shows how the ALSPAC DNA methylation subset was selected.


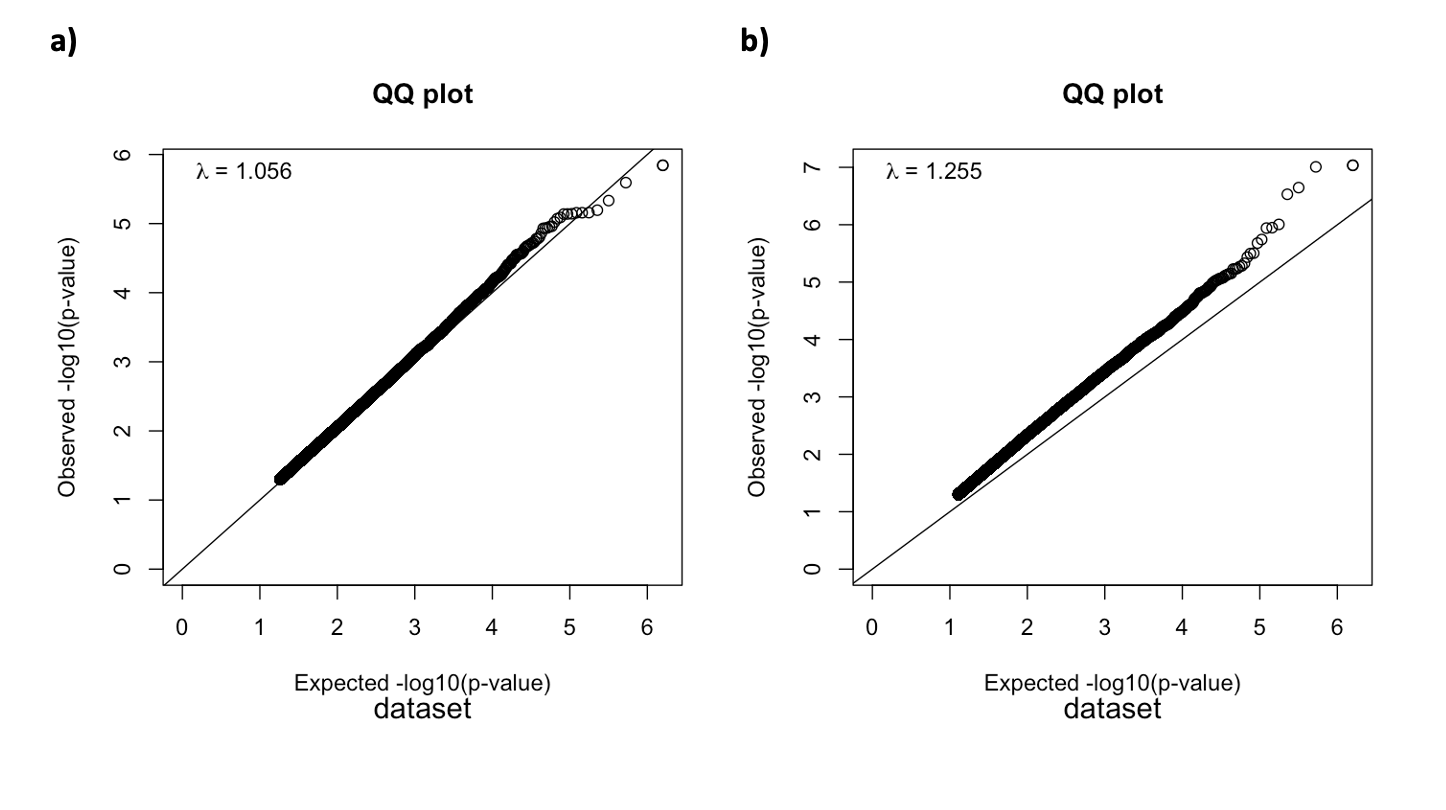


**Supplementary figure 2**: EWAS QQ-plots for model 1 (a) and model 2 i.e, adjusted for steps (b) of the SB EWAS.


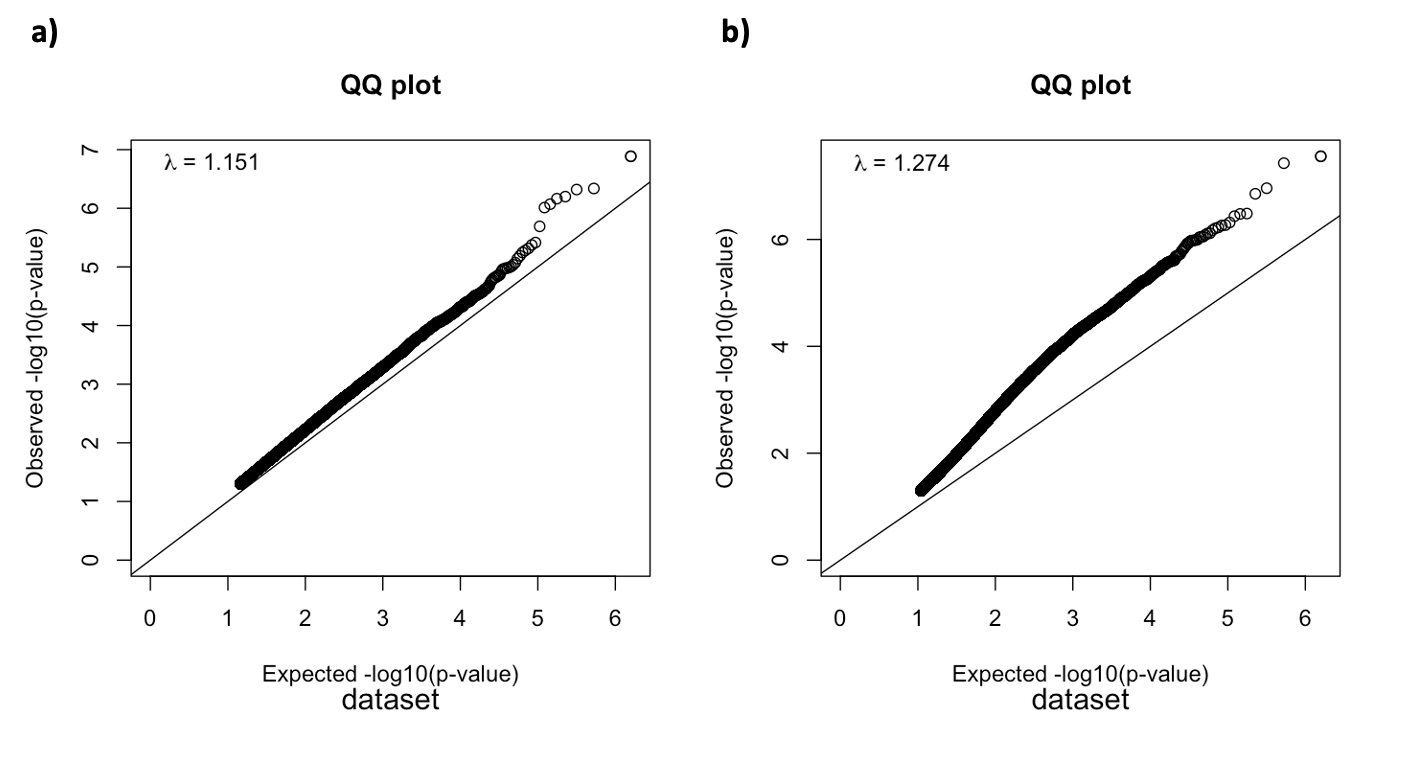


**Supplementary figure 3**: EWAS QQ-plots for model 1 (a) and model 2 i.e, adjusted for steps (b) of the MPA EWAS.
